# Ticks and prevalence of tick-borne pathogens from domestic animals in Ghana

**DOI:** 10.1186/s13071-022-05208-8

**Published:** 2022-03-12

**Authors:** Shirley C. Nimo-Paintsil, Mba Mosore, Seth Offei Addo, Taylor Lura, Janice Tagoe, Danielle Ladzekpo, Charlotte Addae, Ronald E. Bentil, Eric Behene, Courage Dafeamekpor, Victor Asoala, Anne Fox, Chaselynn M. Watters, Jeffrey W. Koehler, Randy J. Schoepp, Hanayo Arimoto, Samuel Dadzie, Andrew Letizia, Joseph W. Diclaro

**Affiliations:** 1 United States Naval Medical Research Unit No. 3, Ghana Detachment, Accra, Ghana; 2 Noguchi Memorial Institute for Medical Research, College of Health Sciences, University of Ghana, Legon, Accra, Ghana; 3 Navy Entomology Center of Excellence, Jacksonville, FL USA; 4 Veterinary Department, Ghana Armed Forces, Accra, Ghana; 5 Navrongo Health Research Center, Navrongo, Upper East Region Ghana; 6 Diagnostic Systems Division, United States Army Medical Research Institute of Infectious Diseases, Fort Detrick, MD USA; 7 Navy Environmental and Preventive Medicine Unit No. 5, San Diego, CA USA; 8 Infectious Diseases Directorate, Naval Medical Research Center, Silver Spring,, MD USA

**Keywords:** Tick-borne pathogens, Livestock, Ghana, West Africa

## Abstract

**Background:**

Ticks are important vectors of various pathogenic protozoa, bacteria and viruses that cause serious and life-threatening illnesses in humans and animals worldwide. Estimating tick-borne pathogen prevalence in tick populations is necessary to delineate how geographical differences, environmental variability and host factors influence pathogen prevalence and transmission. This study identified ticks and tick-borne pathogens in samples collected from June 2016 to December 2017 at seven sites within the Coastal, Sudan and Guinea savanna ecological zones of Ghana.

**Methods:**

A total of 2016 ticks were collected from domestic animals including cattle, goats and dogs. Ticks were morphologically identified and analysed for pathogens such as Crimean-Congo haemorrhagic fever virus (CCHFV), Alkhurma haemorrhagic fever virus (AHFV), *Rickettsia* spp. and *Coxiella burnetii* using polymerase chain reaction assays (PCR) and sequence analysis.

**Results:**

Seven species were identified, with *Amblyomma variegatum* (60%) most frequently found, followed by *Rhipicephalus sanguineus* sensu lato (21%), *Rhipicephalus* spp. (9%), *Hyalomma truncatum* (6%), *Hyalomma rufipes* (3%), *Rhipicephalus evertsi* (1%) and *Rhipicephalus* (*Boophilus*) sp. (0.1%). Out of 912 pools of ticks tested, *Rickettsia* spp. and *Coxiella burnetii* DNA was found in 45.6% and 16.7% of pools, respectively, whereas no CCHFV or AHFV RNA were detected. Co-infection of bacterial DNA was identified in 9.6% of tick pools, with no statistical difference among the ecozones studied.

**Conclusions:**

Based on these data, humans and animals in these ecological zones are likely at the highest risk of exposure to rickettsiosis, since ticks infected with *Rickettsia* spp. displayed the highest rates of infection and co-infection with *C. burnetii*, compared to other tick-borne pathogens in Ghana.

**Graphical Abstract:**

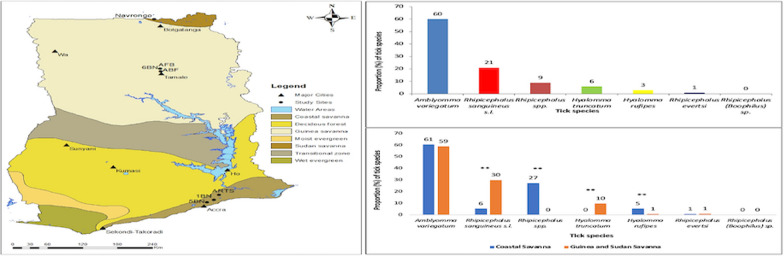

**Supplementary Information:**

The online version contains supplementary material available at 10.1186/s13071-022-05208-8.

## Background

Ticks are important vectors of various pathogenic protozoa, bacteria and viruses that cause morbidity and mortality in humans and animals worldwide [1]. Domestic animals are parasitized by many tick species thereby causing considerable economic loss [2–4]. Human transmission of tick-borne diseases can occur through the bite of an infected tick, exposure to an infected animal, or consuming animal products [5]. Evidence suggests that zoonotic tick-borne diseases are increasing in geographical range, and infection rates are likely to become a major public health threat in the future [6].

Worldwide, ticks serve as important vectors of Crimean-Congo haemorrhagic fever virus (CCHFV) [5], with species of the genus *Hyalomma* considered the principal vectors [7]. Wild and domestic animals such as cattle, sheep and goats play the role of amplifying hosts or reservoirs in the spread of the virus [8]. Although human infections normally occur through tick bites, other possible routes include drinking unpasteurized milk from infected animals and being exposed to blood or tissues from infected individuals or animals infected with the virus [9]. CCHFV is endemic to Africa, the Balkans, the Middle East and Asian countries, with a high case fatality rate [10]. In the Ashanti region of Ghana, which lies within the deciduous forest, the virus has been detected in *Amblyomma variegatum* and *Hyalomma excavatum* ticks collected from cattle at the abattoir, with a seroprevalence rate of 5.7% in animal handlers [11].

Alkhurma haemorrhagic fever virus (AHFV), a tick-borne flavivirus, was originally isolated in 1995 from a patient in Saudi Arabia. Subsequent cases of AHF have been documented in tourists in Egypt, indicating a wider geographical distribution of the virus [12]. Surveillance for this pathogen is supported by the wide distribution of AHFV tick hosts, namely, the soft tick *Ornithodoros savignyi* and the hard tick *Hyalomma dromedarii.* A recent study on AHFV in ticks infesting migratory birds in transit from Africa to Europe and Asia, as well as other cases of seropositivity in Djibouti, could indicate a wider geographical distribution of the virus throughout eastern Africa and possibly the sub-Saharan region [13]. However, the persistence of the virus within tick populations, the role of livestock and the disease transmission process are poorly understood, especially in Ghana and West Africa.

*Coxiella burnetii*, the causative agent of Q fever, is a bacterial pathogen that causes abortion in livestock and is primarily transmitted to humans through infected animal birth products but is also transmitted by ticks [14]. Domestic ruminants represent the most frequent source of animal to human transmission and infection of Q fever [15, 16]. The bacterium is found worldwide with the exception of Antarctica and New Zealand and has been documented in more than 40 species of ticks [17]. However, limited information is available for *C. burnetii* prevalence in sub-Saharan Africa. In Kenya, one study demonstrated *C. burnetii* antibody prevalence of 10–20% in humans, and another showed *C. burnetii* antibody prevalence of 7–57% and 33–34% in domestic cattle and goats, respectively [18, 19]. In Ghana, *C. burnetii* has been detected in children and livestock in three different regions [20–22].

Most rickettsial pathogens are transmitted by ectoparasites during feeding or by scratching crushed infectious arthropods or infectious faeces into the skin. Several pathogenic tick-borne *Rickettsia* species have been found in Africa including in Senegal, Burkina Faso, Cameroon, Mali and Ivory Coast, with human seroprevalence rates ranging from 17 to 36% [23, 24]. In travellers, including military personnel, the most commonly diagnosed rickettsial diseases are usually spotted fever (African tick-bite fever [ATBF]) or typhus groups (murine typhus), but travellers may acquire a wide range of rickettsioses, including emerging and newly recognized species [25].

Military members train and deploy in numerous terrains hospitable to tick populations. Ticks harbour the aforementioned viral and bacterial pathogens that can incapacitate or kill individual troops or cause an outbreak in a region and disrupt force health protection. Surveillance of tick species in Ghana informs potential vector-borne infectious threats for force health protection, improves planning for combatant commands, supports in-country partners and promotes global security.

Vector-borne diseases remain a significant cause of infection throughout the world, but information regarding the risk of tick-borne infections is limited in Africa. Many tick-borne illnesses in humans are associated with domestic animals, particularly livestock [23, 26, 27]. Ghana imports live animals, such as livestock from neighbouring countries. This movement of animals may aid in the transmission of tick-borne disease into Ghana. The spread of disease from livestock trade and migration patterns is compounded by the asymptomatic presentation of some tick-borne diseases in cattle, hindering the ability of inspectors to spot infected animals [16, 28, 29]. Additionally, livestock are commonly allowed free movement to search for water and food. These free-roaming animals have increased exposure to various pathogens that in turn may be transmitted to livestock handlers, veterinarians, abattoir workers and the general population [30]. Ghana is a coastal country bordered by three countries and offers different ecological zones that may influence the distribution of arthropod vectors and their diseases. Thus, this study sought to examine the prevalence of tick-borne pathogens to better understand the public health risk in Ghana and the West African subregion. The scientific benefit includes the augmentation of knowledge regarding the ecology of tick-borne pathogens circulating in the different ecological zones.

## Methods

### Study sites

**Fig. 1:**
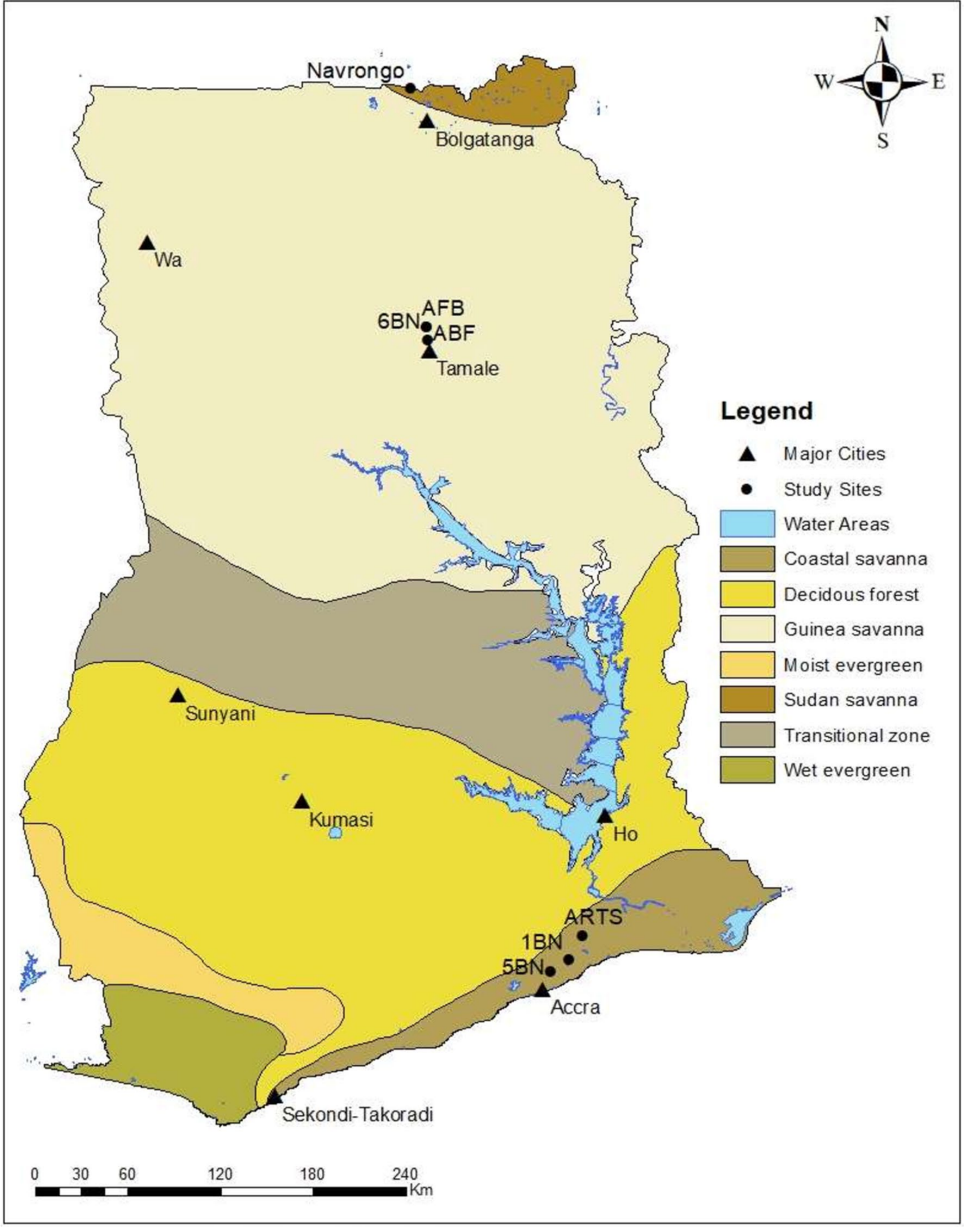
Map of Ghana displaying study sites, and geographical regions. Study sites—[Navrongo, Air Force Base (AF), 6th Battalion Infantry (6 BN), Air Borne Force (ABF), Army Recruit Training School (ARTS), 1st Battalion Infantry (1 BN) and 5th Battalion Infantry (5 BN)]. Tick sampling was conducted in three ecological zones namely; Coastal, Guinea and Sudan savanna between June 2016 and December 2017. The map of Ghana with the geographical regions and study sites was created using ArcGIS^®^ software by Esri (www.esri.com)

One civilian and six military sites within three ecological zones of Ghana were selected for tick collection. Three of the military sites were within the Coastal savanna of Accra; the other three were located in Tamale, all in the Guinea savanna. The one civilian site was located in the Sudan savanna (Fig. 1). Ticks were collected from cattle, goats and dogs in surrounding communities near military bases/camps. Livestock at the study sites were checked for tick infestation.

The Guinea and Sudan savanna sites were chosen because of their proximity to Burkina Faso, Ivory Coast, Togo and Mali. The increased populations of nomadic Fulani herdsmen whose occupation is commercial livestock rearing results in more variety in livestock. However, the influx of livestock from neighbouring countries has the potential of introducing other tick species as well as tick-borne pathogens into the country. In the Coastal savanna sites, most people keep domestic animals such as cats, dogs and chickens alongside cattle for general personal use as opposed to commercial purposes. Herdsmen most often have dogs to accompany livestock during grazing, increasing potential exposure to tick infestation.

### Tick collection

Tick collection was conducted between June 2016 and December 2017. Collection was performed in June and December of 2016 and in March, July, August, September and December of 2017. Verbal consent was sought from animal handlers prior to examining their livestock for ticks. Using blunt forceps, ticks were collected (from the abdomen, neck, internal sides of rear legs, tail and ear) and placed into labelled vials containing RNAlater™ (Qiagen, Germany) ribonucleic acid (RNA) stabilizing reagent. All ticks were morphologically identified with taxonomic keys [31]. Specimens were pooled by species, sex, study site and animal host. Pooled samples consisted of between one to five ticks.

### Nucleic acid extraction and pathogen detection

Pooled ticks were homogenized using Mini-Beadbeater-96 (Biospec, Bartlesville, OK, USA) with lysis buffer and beads of 0.1 mm and 2.0 mm diameter. Nucleic acid was extracted from each pool using the QIAamp Viral RNA Mini Kit (Qiagen, Valencia, CA, USA) following the manufacturer’s instructions [32].

The presence of viral RNA for CCHFV and AHFV was detected using real-time reverse transcriptase polymerase chain reaction (RT-PCR) as described previously [12, 32, 33]. Briefly, 5 ml of extracted nucleic acid was tested in duplicate by real-time RT-PCR using assays for CCHFV and AHFV using the SuperScript One-Step RT-PCR kit (Thermo Fisher Scientific). Synthetic RNAs (BioSyn, Inc., TX, USA) for the CCHFV assay amplicon and the AHFV assay amplicon were used as positive controls, and molecular biology-grade water was used as a negative template control. The positive and negative controls were run on each real-time PCR plate. Fluorescence readings were taken following each real-time PCR cycle, and a sample was considered positive if the quantification cycle (Cq) value was less than 40 cycles. A sample was indeterminate if there was an appropriate curve with a Cq value of greater than 40 cycles, and the sample testing was repeated. The bacterial deoxyribonucleic acid (DNA) of *Rickettsia* spp. and *C. burnetii* was detected separately using quantitative real-time PCR with Platinum TaqDNA Polymerase (Applied Biosystems, Thermo Fisher Scientific, Waltham, MA, USA) assay with sets of primers targeting the gene encoding the 17-kilodalton antigen (17-kDa) of *Rickettsia* DNA and the com1a gene of *C. burnetii*, respectively [33, 34]. Aliquots of double distilled water were included in all PCR runs to detect contamination. The PCR assays were carried out on an ABI 7300 Thermal Cycler (Applied Biosystems, Thermo Fisher Scientific, Waltham, MA, USA).

### Statistical analysis

Tick distribution was described using descriptive statistics with frequency, percentages and bar graphs. The infection rate was estimated using the frequentist approach [35]. For unequal pool size and under the assumption of a perfect test, maximum likelihood (ML) was used to estimate the infection rate. A 95% Wald-type confidence interval was reported for the infection rate. Statistical analysis was done using R version 3.3.0 software. Pearson Chi-square or Fisher’s exact test, where necessary, was used to determine the association between tick species and ecological zones. The association of pooled infection status with animal host and ecological zone was determined using Pearson Chi-square. Statistical significance was set at a *P*-value < 0.005.

## Results

### Species composition of ticks

**Fig. 2:**
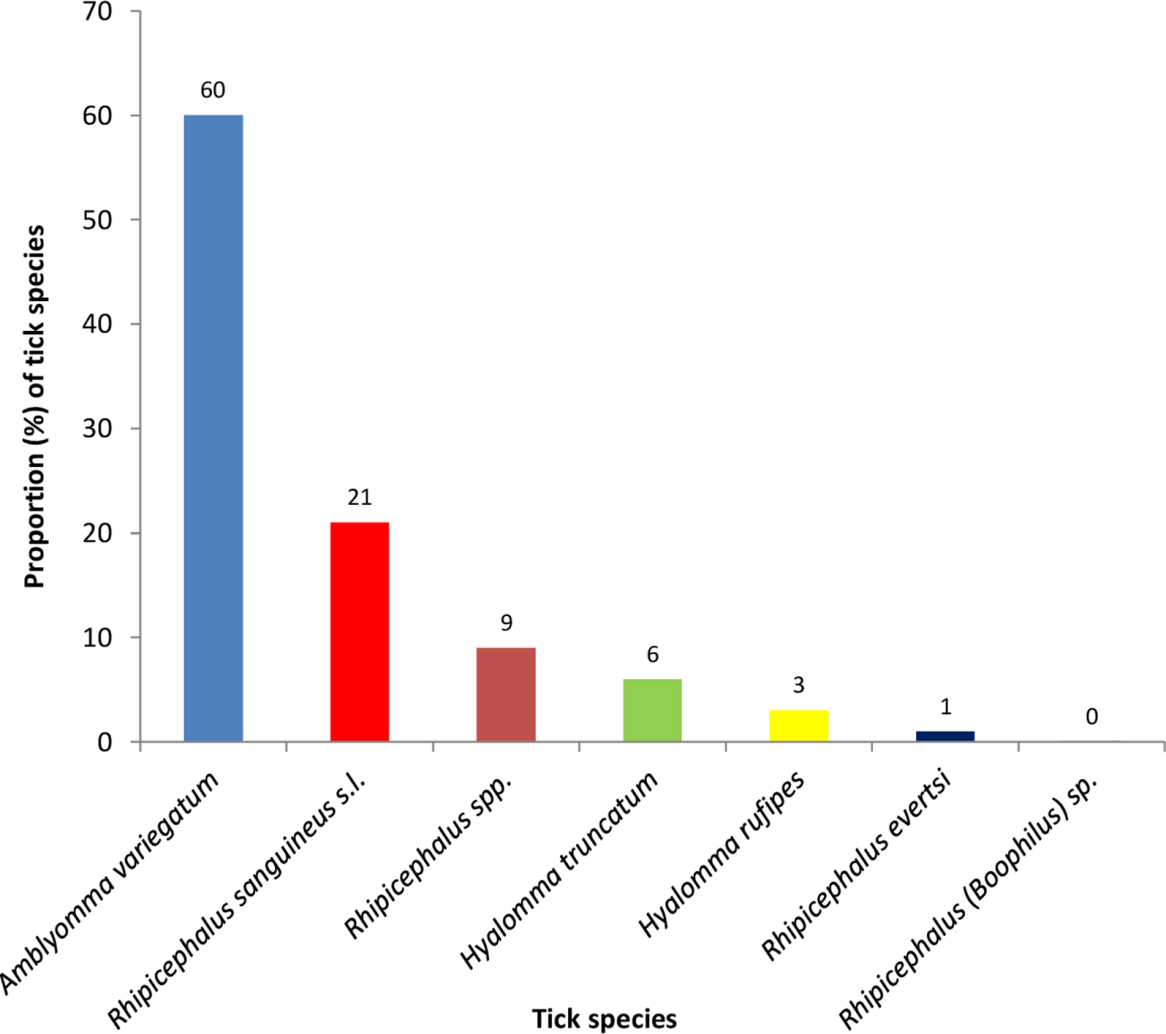
Overall distribution of tick species identified from the three ecological zones. Domestic animals (cattle, goats, dogs) were examined for ticks between June 2016 and December 2017

**Fig. 3:**
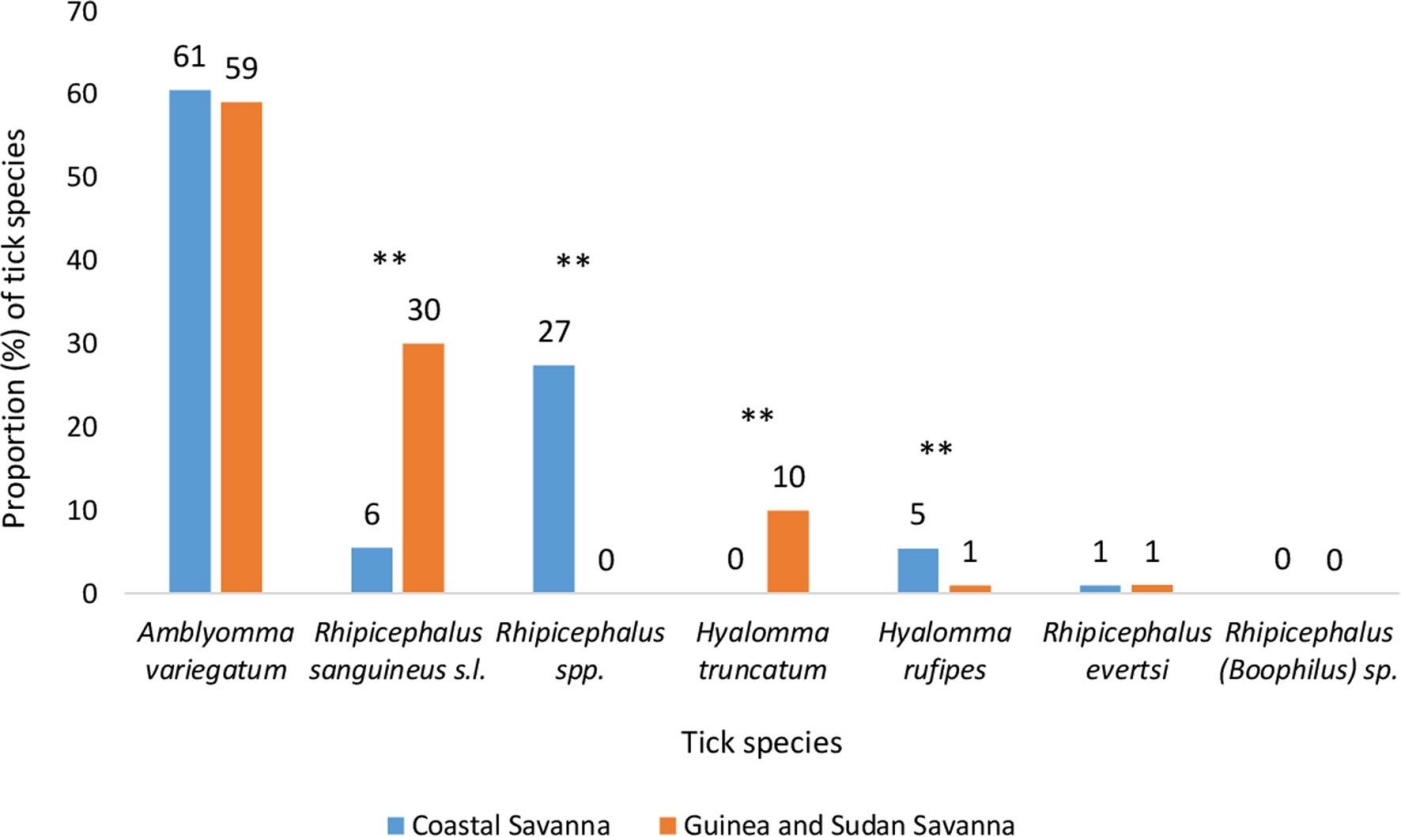
Distribution of tick species identified morphologically from livestock by ecological zone between June 2016 and December 2017 using *χ*^2^ test

A total of 2016 ticks were collected, of which 66 and 34% were males and females, respectively. The majority of ticks sampled (63.3%) were from Guinea and Sudan savanna, while the remaining ticks came from the Coastal savanna of Ghana (Table 1). Seven tick species were identified, with *Amblyomma variegatum* (60%) being the most abundant (Fig. 2). The majority of the *A*. *variegatum* were from cattle (99.8%), whereas *Rhipicephalus sanguineus* sensu lato (s.l.) were found more frequently in dogs (73%) (see Additional file 1). *Rhipicephalus* (*Boophilus*) sp. (0.1%) was only found in the Coastal savanna. Generally, *Rhipicephalus* species occurred more frequently in the Coastal savanna (34%) than in the Guinea and Sudan savanna (31%). Significant differences were recorded in the distribution of *R. sanguineus* s.l. (${\chi }^{2}$= 161.32, *df* = 1, *P* < 0.0001), *Rhipicephalus* spp. (${\chi }^{2}$ = 387.57, *df* = 1, *P* < 0.0001), *Hyalomma truncatum* (${\chi }^{2}$ = 75.59, *df* = 1, *P* < 0.0001) and *Hyalomma rufipes* (${\chi }^{2}$ = 36.93, *df* = 1, *P* < 0.0001) from the three ecological zones of Ghana (Fig. 3).

**Table 1:** Background information of ticks collected

	Total *n* (%)	Coastal savanna *n* (%)	Guinea and Sudan savanna *n* (%)
Animal host			
Cattle	1674 (83.0)	728 (98.5)	946 (74.1)
Dogs	325 (16.1)	11 (1.5)	314 (24.6)
Goats	7 (0.3)	0 (0.0)	7 (0.5)
Sheep	10 (0.5)	0 (0.0)	10 (0.8)
Sex of ticks			
Male	1327 (65.8)	345 (46.7)	982 (76.9)
Female	689 (34.2)	394 (53.3)	295 (23.1)

### Pathogen detection and identification of tick pools

*Coxiella burnetii* was detected at all sites except Navrongo, with a pooled positive rate of 16.7%, whereas a pooled positive rate of 45.6% was recorded for *Rickettsia* spp. and was detected at all seven sites (Table 3). *Coxiella burnetii* was only detected in ticks collected from cattle; however, *Rickettsia* spp. were identified in cattle, goats and dogs. The number of tick pools positive for *C. burnetii* and *Rickettsia* spp. was significant with respect to the animal host and study sites of tick sampling (Table 2). CCHFV and AHFV were not detected in any of the tick pools.

**Table 2:** Association between infection rate of detected pathogens in ticks collected from different hosts and sampling sites using *χ*^2^ test

		Pooled positive, *n* (%)				Pooled positive, *n* (%)			
	Total pooled *n* (%)	*Coxiella burnetii*	*χ*^2^	*df*	*P*-value	*Rickettsia* spp.	*χ*^2^	*df*	*P*-value
Mammalian host			35.61	3	< 0.001		141.63	3	< 0.001
Cattle	763	152 (19.9)				414 (54.3)			
Dogs	141	0 (0.0)				1 (25.0)			
Sheep	4	0 (0.0)				0 (0.0)			
Goats	4	0 (0.0)				1 (0.7)			
Study site			9.35	1	0.002		7.89	1	0.005
* Coastal savanna*	168	66 (39.3)				44 (26.2)			
5 BN	103	10 (9.7)				64 (62.1)			
ART	89	1 (1.1)				35 (39.3)			
1 BN									
*Guinea and Sudan savanna*									
6 BN	99	50 (50.5)				79 (79.8)			
AF	127	23 (18.1)				83 (65.4)			
Navrongo	174	0 (0.0)				5 (2.9)			
ABF	152	2 (1.3)				106 (69.7)			

### Spatial distribution and infection rates

*Rickettsia* spp. were identified at all seven study sites, with Guinea and Sudan savanna ecological zones recording 66% (273/416) of the rickettsial infections in the tick pools (Table 2). While *C. burnetii* was detected in similar pool numbers in the Coastal (77/152) and Guinea (75/152) ecological zones, no infected ticks were identified at the Navrongo site situated in the Sudan savanna ecological zone.

Out of 912 tick pools tested, the overall infection rates for *C. burnetii* and *Rickettsia* spp. were 7.9% (95% CI 6.8–9.2) and 24.5% (95% CI 22.4–26.6), respectively. *Amblyomma variegatum,* the most prevalent species in the tick pools, recorded infection rates of 11.0% (95% CI 9.3–13.0) and 38.6% (95% CI 38.3–42.1) for *C. burnetii* and *Rickettsia* spp., respectively (Table 3).

**Table 3:** Infection rate of tick pools with *Coxiella burnetii* and *Rickettsia* species

				*Coxiella burnetii*			*Rickettsia* spp.		
	No. of pools tested	Min. pool size	Max pool size	No. of positive pools	Mean infection rate (95% CI)		No. of positive pools	Mean infection rate (95% CI)	
*Amblyomma variegatum*	516	1	5	123	11.0 (9.3–13.0)		344	38.6 (38.3–42.1)	
*Rhipicephalus sanguineus* s.l.	200	1	3	3	0.7 (0.2–2.2)		15	3.5 (2.1–5.8)	
*Rhipicephalus* spp.	99	1	5	19	10.5 (6.8–16.0)		22	12.2 (8.2–18.0)	
*Hyalomma truncatum*	56	1	3	5	4.5 (1.9–70.5)		19	17.0 (11.0–25.7)	
*Hyalomma rufipes*	30	1	3	2	3.8 (1.0–14.5)		14	30.2 (18.8–46.2)	
*Rhipicephalus evertsi*	10	1	3	0	0		1	5.1 (0.7–31.4)	
*Rhipicephalus* (*Boophilus*) sp.	1	1	1	0	0		1	100	
Total	912	1	5	152	7.9 (6.8–9.2)		416	24.5 (22.4–26.6)	

Overall co-infection with both *C. burnetii* and *Rickettsia* spp. was identified to be 9.6% (95% CI 8.0–11.7) (Table 4). All co-infections observed were in cattle, with approximately 7.6% being true co-infection in single sample pools. No co-infections were recorded at one site (1st Battalion Infantry) in the Coastal savannah or Navrongo in the Sudan savanna. The majority of co-infections were identified in the Guinea 41% (39/96) and Coastal savanna 31% (30/96) ecological zones. Two of the sites, one in the Coastal and the other in Guinea savanna, recorded co-infections of 6% (*n* = 6) and 1% (*n* = 1), respectively.

**Table 4:** Co-infection of pooled tick species

			*Coxiella burnetii* and *Rickettsia* spp.	
	Min. pool size	Max pool size	No. of co-infection pool	Mean infection rate (95% CI)
*Amblyomma variegatum*	1	3	82	9.6 (7.8–11.8)
*Rhipicephalus sanguineus* s.l.	1	3	0	0
*Rhipicephalus* spp.	1	2	10	19.1 (10.7–32.5)
*Hyalomma truncatum*	1	3	3	9.1 (3.0–25.7)
*Hyalomma rufipes*	1	3	1	3.6 (0.5–23.2)
*Rhipicephalus evertsi*	1	3	0	0
*Rhipicephalus* (*Boophilus*) sp.	1	1	0	0
Total	1	3	96	9.6 (8.0–11.7)

## Discussion

Studies on tick distribution, population and disease presence are sparse in Ghana and are needed to better understand the risk of tick-borne infections within Ghana’s various ecologic zones. The ticks collected in this study reinforce previously recorded distributions of tick species in Ghana [36]. Of the ticks collected, several are associated with the transmission of human pathogens. *Amblyomma variegatum* is widely distributed throughout Ghana and has been collected from both domestic and wild animals [36–38]. Therefore, it is predictable that in this study, *A. variegatum* was the most abundant tick species collected. Generally, in the ecozones studied, *A. variegatum* seemed to thrive well. This distribution and abundance are of note, however, as *A. variegatum* has been implicated as a vector of diseases such as ATBF and CCHF [39, 40]. The *Hyalomma* spp. are known vectors for CCHFV, and *R. sanguineus* s.l. has been implicated as a vector for some *Rickettsia* spp. including those in the spotted fever group [4, 5, 39]. Currently, ATBF has not been recorded in Ghana; however many neighbouring countries have reported cases. The presence of *Hyalomma* spp. and *R. sanguineus* s.l. at the study sites could support the transmission of the pathogens that cause CCHFV and rickettsioses. Nonetheless, CCHFV was not identified in any of the *Hyalomma* spp. and this could be due to low sample size or that they are not vectors of the disease in Ghana.

Previous studies have found that tick species, including *H. rufipes*, *H. truncatum* and *R. evertsi*, are found almost exclusively on domestic ruminants [36–38]. This observation could be explained by host preference and/or environmental preference of these tick species as some studies have found that these factors impact tick distribution in Ghana [37]. Ticks taken from dogs in this study were from communities in which the livestock were present and normally accompany the domestic ruminants to grazing fields. It is unclear whether this is a host preference or simply that the domestic dogs are within the same habitat as the livestock, and therefore the ticks were feeding opportunistically.

*Coxiella burnetii* (16.7%) and *Rickettsia* spp. (45.6%) were detected in the pooled tick samples collected in this study. For both *C. burnetii* and *Rickettsia* spp., the infection rate was highest in the tick species that are often associated with the transmission of Q fever and rickettsial diseases, respectively, including *A. variegatum*, *Rhipicephalus* spp., *H. truncatum* and *H. rufipes* [7, 39–41]. *Coxiella burnetii* is often asymptomatic in cattle, barring some reproductive issues, and causes chronic infections [16]. Studies have reported that *C. burnetii* is at particularly high concentrations during times of parturition in infected animals, possibly increasing the risk of transmission to anyone handling or in contact with newborns, placental tissues and amniotic fluids [42]. Cases of Q fever have been reported in Ghana, including an outbreak in the Volta region associated with interaction with small ruminants [20, 22].

In the present study, approximately 25% of the ticks tested were infected with *Rickettsia* spp. The highest rate of infection was in *A. variegatum*, possibly due to the wide distribution of *A. variegatum* throughout Ghana. A few cases of rickettsial diseases have been reported in Ghana. Past studies have documented the detection of *Rickettsia* spp. in ticks and humans in Ghana, though they were not further identified to species [43, 44]. It is possible that *R. africae* or other *Rickettsia* spp. pathogens are present in Ghana, which highlights the importance of further research and identification of all *Rickettsia* spp. detected. Additionally, since livestock are allowed to roam freely, there are increased opportunities for ticks to be distributed to novel areas of Ghana and consequently potential transmission of tick-borne pathogens such as *R. africae* to humans.

Four of the seven tick species were positive for *C. burnetii* and *Rickettsia* spp. co-infection. Additionally, the *Rhipicephalus* spp. pools had the highest rate of co-infection with these two pathogens. However, actual co-infection in single sample pools accounted for 7.6%. Co-infection of pathogens within ticks is fairly common [45–47]. Furthermore, co-infection of *C. burnetii* and *Rickettsia* spp. has been recorded in several tick species and is often the most common combination found [4, 45, 48]. These co-infections may be the result of subsequent blood meals, feeding on a co-infected host or co-feeding with other infected ticks [4, 49]. Multiple pathogen co-infection of ticks may lead to many complications, including further spread and distribution of diseases and complications of clinical diagnosis. Multiple tick pathogen co-infection in humans may complicate clinical diagnosis and, subsequently, proper treatment [4, 50]. Pathogens may require different treatments; therefore, mis- or undiagnosed co-infections may result in prolonged illness in patients [4, 50].

Tick collection was not conducted periodically throughout the year, and as a result, the study did not associate tick distribution with the dry and wet seasons of Ghana. The study is also limited in selecting sampling sites and, therefore, could not cover all the Ghanaian ecological zones to provide a better representation of tick and tick-borne pathogens in the entire country. Another limitation of this study is that the majority of the co-infections detected were in pooled samples of two or three ticks, and therefore, one cannot differentiate whether these are actual co-infections or whether multiple ticks within a pooled sample each carried one pathogen.

## Conclusions

Among approximately 2000 ticks mostly obtained from cattle that were examined from three ecologic zones in Ghana, 2016 ticks were identified, with 16.7% and 45.6% rates of *Coxiella* and *Rickettsia* species, respectively. As the world becomes more globalized and trade between countries increases, it is imperative that research on current tick species and possible disease introduction to new areas be monitored. Continued surveillance and pathogen testing are important to track the possible introduction of new tick pathogens entering the country. As the importation of livestock increases to meet the demand for dairy and meat products, so does the likelihood for the importation of new diseases.

## Supplementary Information

**Additional file 1**. Distribution of tick species collected from livestock during the study period.

## Data Availability

Supporting data for the conclusions of this article are included within the article and its additional files. The raw datasets used and analysed during this study are available upon reasonable request.

## Abbreviation

CI: Confidence interval

## References

[1] Gondard M, Cabezas-Cruz A, Charles RA, Vayssier-Taussat M, Albina E, Moutailler S (2017). Ticks and tick-borne pathogens of the Caribbean: current understanding and future directions for more comprehensive surveillance. Front Cell Infect Microbiol.

[2] Rajput ZI, Hu SH, Chen WJ, Arijo AG, Xiao CW (2006). Importance of ticks and their chemical and immunological control in livestock. J Zhejiang Univ Sci B.

[3] Vesco U, Knap N, Labruna MB, Avsic-Zupanc T, Estrada-Pena A, Guglielmone AA (2011). An integrated database on ticks and tick-borne zoonoses in the tropics and subtropics with special reference to developing and emerging countries. Exp Appl Acarol.

[4] Reye AL, Arinola OG, Hubschen JM, Muller CP (2012). Pathogen prevalence in ticks collected from the vegetation and livestock in Nigeria. Appl Environ Microbiol.

[5] Telmadarraiy Z, Chinikar S, Vatandoost H, Faghihi F, Hosseini-Chegeni A (2015). Vectors of Crimean Congo hemorrhagic fever virus in Iran. J Arthropod-Borne Dis..

[6] Estrada-Pena A, de la Fuente J (2014). The ecology of ticks and epidemiology of tick-borne viral diseases. Antivir Res.

[7] Bente DA, Forrester NL, Watts DM, McAuley AJ, Whitehouse CA, Bray M (2013). Crimean-Congo hemorrhagic fever: history, epidemiology, pathogenesis, clinical syndrome and genetic diversity. Antivir Res.

[8] Hawman DW, Feldmann H (2018). Recent advances in understanding Crimean-Congo hemorrhagic fever virus. F1000Research..

[9] Lwande OW, Irura Z, Tigoi C, Chepkorir E, Orindi B, Musila L (2012). Seroprevalence of Crimean Congo hemorrhagic fever virus in Ijara District, Kenya. Vector Borne Zoonotic Dis.

[10] Raabe VN (2020). Diagnostic testing for Crimean-Congo hemorrhagic fever. J Clin Microbiol.

[11] Akuffo R, Brandful JA, Zayed A, Adjei A, Watany N, Fahmy NT (2016). Crimean-Congo hemorrhagic fever virus in livestock ticks and animal handler seroprevalence at an abattoir in Ghana. BMC Infect Dis.

[12] Carletti F, Castilletti C, Di Caro A, Capobianchi MR, Nisii C, Suter F (2010). Alkhurma hemorrhagic fever in travelers returning from Egypt, 2010. Emerg Infect Dis.

[13] Hoffman T, Lindeborg M, Barboutis C, Erciyas-Yavuz K, Evander M, Fransson T (2018). Alkhurma hemorrhagic fever virus RNA in *Hyalomma rufipes* ticks infesting migratory birds, Europe and Asia Minor. Emerg Infect Dis.

[14] Vanderburg S, Rubach MP, Halliday JE, Cleaveland S, Reddy EA, Crump JA (2014). Epidemiology of *Coxiella burnetii* infection in Africa: a OneHealth systematic review. PLoS Negl Trop Dis.

[15] Marrie TJ (1990). Q fever—a review. Can Vet J La revue veterinaire canadienne..

[16] Maurin M, Raoult D (1999). Q fever. Clin Microbiol Rev.

[17] Hilbink F, Penrose M, Kovacova E, Kazar J (1993). Q fever is absent from New Zealand. Int J Epidemiol.

[18] Gossler R, Hunermund G (1973). Serological studies on cattle in catchment area of Kabete (Kenia). 2. Determination of antibodies against *Mycobacterium paratuberculosis*, *Brucella*, *Salmonella*, *Pasteurella multocida*, *Listeria* and *Leptospira*. Berl Munch Tierarztl Wochenschr.

[19] Vanek E, Thimm B (1976). Q fever in Kenya. Serological investigations in man and domestic animals. East Afr Med J.

[20] Kobbe R, Kramme S, Kreuels B, Adjei S, Kreuzberg C, Panning M (2008). Q fever in young children, Ghana. Emerg Infect Dis.

[21] Adu-Addai B, Koney EB, Addo P, Kaneene J, Mackenzie C, Agnew DW (2012). Importance of infectious bovine reproductive diseases: an example from Ghana. Vet Rec.

[22] Johnson SAM, Kaneene JB, Asare-Dompreh K, Tasiame W, Mensah IG, Afakye K (2019). Seroprevalence of Q fever in cattle, sheep and goats in the Volta region of Ghana. Vet Med Sci.

[23] Parola P, Inokuma H, Camicas JL, Brouqui P, Raoult D (2001). Detection and identification of spotted fever group Rickettsiae and Ehrlichiae in African ticks. Emerg Infect Dis.

[24] Mediannikov O, Diatta G, Fenollar F, Sokhna C, Trape JF, Raoult D (2010). Tick-borne rickettsioses, neglected emerging diseases in rural Senegal. PLoS Negl Trop Dis.

[25] Leshem E, Meltzer E, Schwartz E (2011). Travel-associated zoonotic bacterial diseases. Curr Opin Infect Dis.

[26] Kjemtrup AM, Conrad PA (2000). Human babesiosis: an emerging tick-borne disease. Int J Parasitol.

[27] Spengler JR, Bergeron E, Rollin PE (2016). Seroepidemiological studies of Crimean-Congo hemorrhagic fever virus in domestic and wild animals. PLoS Negl Trop Dis.

[28] Chinikar S, Ghiasi SM, Hewson R, Moradi M, Haeri A (2010). Crimean-Congo hemorrhagic fever in Iran and neighboring countries. J Clin Virol.

[29] Appannanavar SB, Mishra B (2011). An update on Crimean Congo hemorrhagic fever. J Glob Infect Dis..

[30] Amissah-Reynolds PK (2020). Zoonotic risks from domestic animals in Ghana. Int J Pathogen Res.

[31] Walker AR, Bouattour A, Camicas J-L, Estrada-Peña A, Horak IG, Latif AA, et al. Ticks of domestic animals in Africa: a guide to identification of species. Bioscience Reports University of Edinburgh 2003.

[32] Garrison AR, Alakbarova S, Kulesh DA, Shezmukhamedova D, Khodjaev S, Endy TP (2007). Development of a TaqMan minor groove binding protein assay for the detection and quantification of Crimean-Congo hemorrhagic fever virus. Am J Trop Med Hyg.

[33] Koehler JW, Delp KL, Hall AT, Olschner SP, Kearney BJ, Garrison AR (2018). Sequence optimized real-time reverse transcription polymerase chain reaction assay for detection of Crimean-Congo hemorrhagic fever virus. Am J Trop Med Hyg.

[34] Jiang J, Stromdahl EY, Richards AL (2012). Detection of *Rickettsia parkeri* and *Candidatus* Rickettsia andeanae in *Amblyomma maculatum* Gulf Coast ticks collected from humans in the United States. Vector Borne Zoonotic Dis.

[35] Mc VMLL, Branscum AJ, Collins MT, Gardner IA (2008). Frequentist and Bayesian approaches to prevalence estimation using examples from Johne's disease. Anim Health Res Rev.

[36] Ntiamoa-Baidu Y, Carr-Saunders C, Matthews BE, Preston PM, Walker AR (2004). An updated list of the ticks of Ghana and an assessment of the distribution of the ticks of Ghanaian wild mammals in different vegetation zones. Bull Entomol Res.

[37] Ntiamoa-Baidu Y, Carr-Saunders C, Matthews BE, Preston PM, Walker AR (2005). Ticks associated with wild mammals in Ghana. Bull Entomol Res.

[38] Walker AR, Koney EB (1999). Distribution of ticks (Acari: Ixodida) infesting domestic ruminants in Ghana. Bull Entomol Res.

[39] de la Fuente J, Estrada-Pena A, Venzal JM, Kocan KM, Sonenshine DE (2008). Overview: ticks as vectors of pathogens that cause disease in humans and animals. Front Biosci J Virtual Libr.

[40] Dantas-Torres F, Chomel BB, Otranto D (2012). Ticks and tick-borne diseases: a One Health perspective. Trends Parasitol.

[41] Jongejan F, Uilenberg G (2004). The global importance of ticks. Parasitology.

[42] Plummer PJ, McClure JT, Menzies P, Morley PS, Van den Brom R, Van Metre DC (2018). Management of *Coxiella burnetii* infection in livestock populations and the associated zoonotic risk: a consensus statement. J Vet Intern Med.

[43] Andoh M, Sakata A, Takano A, Kawabata H, Fujita H, Une Y (2015). Detection of *Rickettsia* and *Ehrlichia* spp. in ticks associated with exotic reptiles and amphibians imported into Japan. PLoS ONE.

[44] Amoako N, Duodu S, Dennis FE, Bonney JHK, Asante KP, Ameh J (2018). Detection of dengue virus among children with suspected malaria, Accra, Ghana. Emerg Infect Dis.

[45] Nooroong P, Trinachartvanit W, Baimai V, Ahantarig A (2018). Phylogenetic studies of bacteria (*Rickettsia*, *Coxiella*, and *Anaplasma*) in *Amblyomma* and *Dermacentor* ticks in Thailand and their co-infection. Ticks Tick-Borne Dis..

[46] Raileanu C, Moutailler S, Pavel I, Porea D, Mihalca AD, Savuta G (2017). Borrelia diversity and co-infection with other tick borne pathogens in ticks. Front Cell Infect Microbiol.

[47] Moutailler S, Valiente Moro C, Vaumourin E, Michelet L, Tran FH, Devillers E (2016). Co-infection of ticks: the rule rather than the exception. PLoS Negl Trop Dis.

[48] Clay K, Klyachko O, Grindle N, Civitello D, Oleske D, Fuqua C (2008). Microbial communities and interactions in the lone star tick, *Amblyomma americanum*. Mol Ecol.

[49] Randolph SE, Gern L, Nuttall PA (1996). Co-feeding ticks: epidemiological significance for tick-borne pathogen transmission. Parasitol Today.

[50] Swanson SJ, Neitzel D, Reed KD, Belongia EA (2006). Coinfections acquired from Ixodes ticks. Clin Microbiol Rev.

