# Supplementary material for: Ticks and prevalence of tick-borne pathogens from domestic animals in Ghana
Source: Parasit Vectors. 2022 Mar 12;15:86. doi: 10.1186/s13071-022-05208-8 (PMC8917784; doi:10.1186/s13071-022-05208-8)
Supplement: Supplementary file 1 — Additional file 1. Distribution of tick species collected from livestock during the study period. [file 13071_2022_5208_MOESM1_ESM.docx]

**Additional file 1**

**Distribution of tick species collected from livestock during study period**

| **Tick species** | **Cattle *n* (%)** | **Dog *n* (%)** | **Goat *n* (%)** | **Sheep *n* (%)** |
| --- | --- | --- | --- | --- |
| ***Amblyomma variegatum*** | **1205 (99.8)** | **2 (0.2)** | **0 (0.0)** | **0 (0.0)** |
| ***Hyalomma truncatum*** | **114 (100)** | **0 (0.0)** | **0 (0.0)** | **0 (0.0)** |
| ***Hyalomma rufipes*** | **53 (100)** | **0 (0.0)** | **0 (0.0)** | **0 (0.0)** |
| ***Rhipicephalus sanguineus* s.l.** | **112 (26.0)** | **312 (72.6)** | **6 (1.4)** | **0 (0.0)** |
| ***Rhipicephalus* (*Boophilus*) sp.** | **1 (100)** | **0 (0.0)** | **0 (0.0)** | **0 (0.0)** |
| ***Rhipicephalus evertsi*** | **10 (47.6)** | **0 (0.0)** | **1 (4.8)** | **10 (47.6)** |
| ***Rhipicephalus* spp.** | **179 (94.2)** | **11 (5.8)** | **0(0.0)** | **0 (0.0)** |
